# The MoCA dataset, kinematic and multi-view visual streams of fine-grained cooking actions

**DOI:** 10.1038/s41597-020-00776-9

**Published:** 2020-12-15

**Authors:** Elena Nicora, Gaurvi Goyal, Nicoletta Noceti, Alessia Vignolo, Alessandra Sciutti, Francesca Odone

**Affiliations:** 1 MaLGa Center - DIBRIS - Università di Genova, Genova, Italy; 2 CONTACT Unit - Istituto Italiano di Tecnologia, Genova, Italy

**Keywords:** Computational science, Scientific data

## Abstract

MoCA is a bi-modal dataset in which we collect Motion Capture data and video sequences acquired from multiple views, including an ego-like viewpoint, of upper body actions in a cooking scenario. It has been collected with the specific purpose of investigating view-invariant action properties in both biological and artificial systems. Besides that, it represents an ideal test bed for research in a number of fields – including cognitive science and artificial vision – and application domains – as motor control and robotics. Compared to other benchmarks available, MoCA provides a unique compromise for research communities leveraging very different approaches to data gathering: from one extreme of action recognition in the wild – the standard practice nowadays in the fields of Computer Vision and Machine Learning – to motion analysis in very controlled scenarios – as for motor control in biomedical applications. In this work we introduce the dataset and its peculiarities, and discuss a baseline analysis as well as examples of applications for which the dataset is well suited.

## Abstract (summary)

**Table:** 

Measurement(s)	body movement coordination trait • food cooking process
Technology Type(s)	motion capture • digital camera
Factor Type(s)	viewpoint

Machine-accessible metadata file describing the reported data: 10.6084/m9.figshare.13207916

## Background & Summary

The Multiview Cooking Actions dataset (MoCa) is a bi-modal dataset acquired to understand motion recognition skills and view-invariance properties of both biological and artificial perceptual systems.

Unlike other recently proposed datasets, where actions and activities are observed in highly unconstrained scenarios^1,2^, our dataset has been acquired in a set-up designed to achieve a compromise between *precision* and *naturalness* of the movement. Such properties make our dataset an ideal test bed for a number of fields and related research questions, among which it is worth mentioning the following:

- *Cognitive science*, in particular for the study of action understanding in humans and the design of artificial intelligence simulations. Different problems can be tackled, ranging from the estimation of actions similarity, to the detection of action primitives, and the identification of taxonomies. A further, challenging, research goal for which our dataset is appropriate is investigating motion properties allowing for actions anticipation and the understanding of their final goal
- *Motor control*, a domain where marker-based approaches constitute the gold standard. However, there is a growing interest in markerless methods which can guarantee naturalness of movements, and thus a higher reliability of the analysis. In this sense, our dataset can serve as a basis for a comparative analysis on the quality of marker-less methods as opposed to classical marker-based ones.
- *View-invariance in action recognition*, an open question of cognitive science and artificial intelligence systems. The combination of multi-view videos and the corresponding position of anatomical features in the 3D space, may be a useful test bed to assess the descriptive power of view-invariant representations.
- *Collaborative robotics*, where a fast comprehension of what the partner is doing and when it is the right moment to act is a fundamental ability. In this respect, our dataset provides a collection of daily life activities, which could serve both in the context of action recognition from the robot camera and in the perspective of generating appropriate robot motions.

## On the Uniqueness of the Dataset

We report in Table 1 a comparison between the MoCA and existing datasets for motion understanding tasks. We pay particular attention to considering visual data and fields related to potentials usages of the MoCA.

**Table 1:** A comparison of the MoCA dataset with existing benchmarks: HMDB^3^, Activitynet^1^, HACS^4^, Kinectics-700^2^, UCF 101^5^, MPII Cooking 2^6^, EPIC-Kitchens^11^, You Cook 2^35^, Arbitrary view^7^, IXMAS^8^, NUCLA^9^, NTU^10^, Schreiber & Moissenet^12^, Fukuchi *et al*.^13^, UE-HRI^36^, CMU-MMAC^37^, TUM Kitchen^38^, Ego Yale^39^.

Dataset	Visual sensors	View(s) Setup	Body part	Envir.	Acquisition Conditions	Annotated Task
^3^	RGB	FV^a^	Full/Upper	Clutt.	Web	Action Rec.
^1^	RGB	FV	Full/Upper	Clutt.	Web	Activity Rec.
^4^	RGB	FV	Full/Upper	Clutt.	Web	Action Rec.
						Action det.
^2^	RGB	FV	Full/Upper	Clutt.	Web	Action Rec.
^5^	RGB	FV	Full/Upper	Clutt.	Web	Action Rec.
^6^	RGB	1 V^b^	Full/Upper	Clutt.	LabFM	Activity Rec.
^11^	RGB	Ego	Arms	Clutt.	LabFM	Activity Rec.
						Object Rec.
^35^	RGB	FV	(Mostly) upper	Clutt.	Web	Activity Rec.
						Object Rec.
^7^	RGB-D*	6 V	Upper	Clean	LabPA	Action Rec.
	Skeleton*	CVV				
^8^	RGB*	5 V	Full	Clean	LabPA	Action Rec.
^9^	RGBD*	3 V	Full	Both	LabPA	Action Rec.
	Skeleton*					
^10^	RGBD*	3 V^c^	Full	Clean	LabPA	Action Rec.
	Skeleton*					
^12^	Skeleton	—	Full	—	LabGait	Gait analysis
^13^	Skeleton	—	Legs	—	LabGait	Gait analysis
^36^	RGB-D	FV	Upper	Clean	LabFM	Human engag.
^37^	RGB*	5 V	Full/Upper	Clutt.	LabFM	Activity Rec.
	Skeleton*					
^38^	RGB*	4 V	Full	Clutt.	LabFM	Markerless Motion
	Skeleton*					Analysis
						Activity Rec.
						Activity det.
^39^	RGB	Ego	Arms	Clutt.	LabFM	Grasp Analysis
MoCA	RGB*	3 V	Upper	Clean	LabPA	Motion Analysis
	Skeleton*					Action Primitive Det.
						Action Rec.

The column **View(s) setup** reports info on the setup referring to the camera setup, that may include different fixed cameras (nV, where n is the number of cameras), may have no specific constraint on the mutual position between camera and subject (we named *Free Viewpoint* - FV), or may consider the use of a wearable ego camera (referred to as Ego). In addition, one the benchmarks also includes a continuous varying view (CVV). In case of multiple views and/or visual modalities, an refers to the fact the streams have been acquired synchronously, meaning that all the visual sensors observed the very same dynamic event. The column **Environment** indicates whether the acquisitions have been performed in a cluttered scene or collected from the web (in both cases referred to as Clutt.) or acquired with a clean background to focus on specific aspects of the analysis (clean). In column **Acquisitions conditions** we report information on the fact videos have been collected online (Web) or in a laboratory, considering predefined actions (LabPA), free movements (LabFM), or more specifically gaits (LabGait). NOTES ^a^Actions annotated according to 4 course views (front, back, left, right)^b^Among the 7 views considered, only one is fully available. ^c^Height and distance of the 3 cameras have been varied to collect acquisitions from a richer set of viewpoints.

Datasets recently proposed for benchmarking action and activity recognition methods are mostly acquired in unconstrained environments or collected from the web^1–6^. In particular, multi-view^7–10^ and arbitrary view^7^ datasets are often acquired with the goal of designing action recognition methods robust to view-point changes. Another specific type of research question is the one involving ego-vision, here the interest is growing but the availability of ad hoc datasets is still limited^11^. To the best of our knowledge, to date, there is no multi-view dataset of motion activities which is also incorporating an egocentric view point.

In the context of motor control, particularly in the biomedical field, the use of markers represents a gold standard, and this reflects of the main characteristics of the datasets proposed to the purpose^12,13^, very precise in terms of measurements, but with a limited naturalness. Usually these datasets consider very specific action classes, especially gait.

MoCA represents a unique compromise for a visual dataset between the very different approaches to data gathering of research communities: from one extreme of action recognition in the wild – the common practice nowadays in the fields of Computer Vision and Machine Learning – to motion analysis in very controlled scenarios – as in biomedical applications. Indeed, MoCA provides multi-modal sequences offering different levels precision and richness (very precise but sparse when derived from the motion capture system, dense but noisier when derived from videos), a high variability in terms of observed actions and motion patterns of different granularity, including both large arms motions and fine fingers movements. Moreover, the availability of multiple synchronised videos of the same instance of a dynamic event enables the investigation of view-invariant motion properties at a level of detail rarely provided by other datasets. Lastly, the selection of the specific views adopted is inspired by interactive contexts, where the agent - be it a human or a robot - has in general a view in first perspective (ego) of its own actions and observes its partners most often collaborating with it either in front of it, or at its side. The combination of the three camera streams, and their 2D perspectives, with the 3D information coming from motion capture data is particularly suited to all applications meant to address action understanding for interactive purposes, supporting - from the perceptual side - cross-view action mapping and - from the motor side - the generation of appropriate arm actions.

## Methods

In this section we highlight the main properties of our data collection. We start describing the design of the study and the setup, with its main technical characteristics. Then, we will discuss the pre-processing we applied to the data for cleaning and synchronisation purposes.

### Study design

The literature on human motion understanding from visual data has grown considerably over the years, influenced by different fields of application, tasks, types of data and feature representation. For these reasons, the problem has been shaped and referred to in a variety of ways, as action classification or recognition^14^, gesture recognition^15^, activity classification^16^. There is no commonly accepted definition of such tasks. For instance, the terms gesture and motion primitive are often used interchangeably, as well as the concept of action and activity. At the same time their use may reflect slightly different nuances of the problem. If we consider a cooking scenario, we can imagine an activity being the whole process of preparing a recipe or part of it, an action being an intermediate step like “mix all the ingredients together” or “peel and slice the apples” and a gesture being “reaching for the apple”.

More formally, according to^17^ actions can be naturally decomposed in phases: each action phase corresponds to a primitive gesture, characterized by changes in sub-goals and related to mechanical events^18^. An activity can be hierarchically defined as a sequence of actions.

An additional aspect that needs to be considered is the definition of **action instance**: with instance (or segment) we refer to an entire action, associated with a corresponding subgoal, that may be composed by one or more gestures, depending on the action itself. Roughly speaking, it may be defined as a motion “portion” after which the subgoal has been met.The action of “beating eggs”, for example, may be intended as a repetition of circular gestures, whereas an action like “rolling the dough” may be considered like the union of two atomic gestures (a movement forward and a movement backward).

With this ideal formulation in mind, we identified as an appropriate context, a *cooking scenario*, a compromise between control of the setup and variability of possible actions type. We then selected a variety of upper-body actions, with the specific aim of providing a good variability in terms of spatio-temporal properties and complexity. Indeed, the range of actions presents significant diversity in terms of motion granularity – as they may involve the movement of fingers, hands or the entire arms – and dynamics – including both slow and faster movements. Also, they may involve the use of one or two arm(s) and possibly the use of tools might require application of a variety of forces. Only one volunteer has been involved in the acquisitions. On this aspect, we notice that our main motivations for the acquisition of the dataset is related with the assessment of movements variability across different actions type and with the analysis of their main components. For these reasons, we put our attention on the richness of actions portfolio, while controlling the complexity of the problem with respect to other sources of variability, being the one related with the presence of many actors one of them.

We report in Table 2 the list of actions we considered in our data collection, which we believe provides a good test-bed scenario, accounting for all the considerations above. To enforce this observation, we show in the table some objective properties about the actions and their structure. In particular, we defined two distinct possible categorizations of our actions. The first one refers to the composition of an action in terms of motion primitives, identifying three possible cases:

**Table 2:** List of actions included in the MoCA, with associated main characteristics (see the text for details on the categorizations).

Action		Structure					Objects	Arms
		P1	P2	P3	S	M		
1	Shred a carrot		x			x	n	1
2	Cut the bread			x	x		y	1
3	Clean a dish		x			x	y	1
4	Eat		x		x		n	1
5	Beat eggs		x			x	y	1
6	Squeeze a lemon		x		x		y	1
7	Mince with a crescent		x			x	y	2
8	Mix in a bowl		x			x	y	1
9	Open a bottle			x	x		y	2
10	Turn the pancake			x	x		y	1
11	Pestle		x		x		y	1
12	Pour water in containers			x		x	y	1
13	Pour water in a mug			x	x		y	1
14	Reach an object	x			x		n	1
15	Roll the dough		x			x	y	2
16	Wash the salad		x			x	y	1
17	Salt			x	x		y	1
18	Spread cheese on bread			x	x		y	2
19	Clean the table		x			x	y	1
20	Transport an object	x			x		y	1

**P1** An action composed by a single primitive

**P2** An action composed by a sequence of the same primitive

**P3** An action composed by a sequence of different primitives

The second categorization refers to the notion of action instance in relation to the achievement of a goal. In this sense we may identify two different actions classes:

**S** “Single shot” actions, for which a single instance is enough to reach the goal (e.g., open the bottle or transporting an object)

**M** “Multiple” actions, for which a sequence of primitive gestures, of variable length, is required (e.g., grating a carrot or mixing ingredients)

The last columns of the table refers to the presence of objects (for manipulating actions) and the fact that the action may involve both arms or just one.

### Data acquisition

**Fig. 1:**
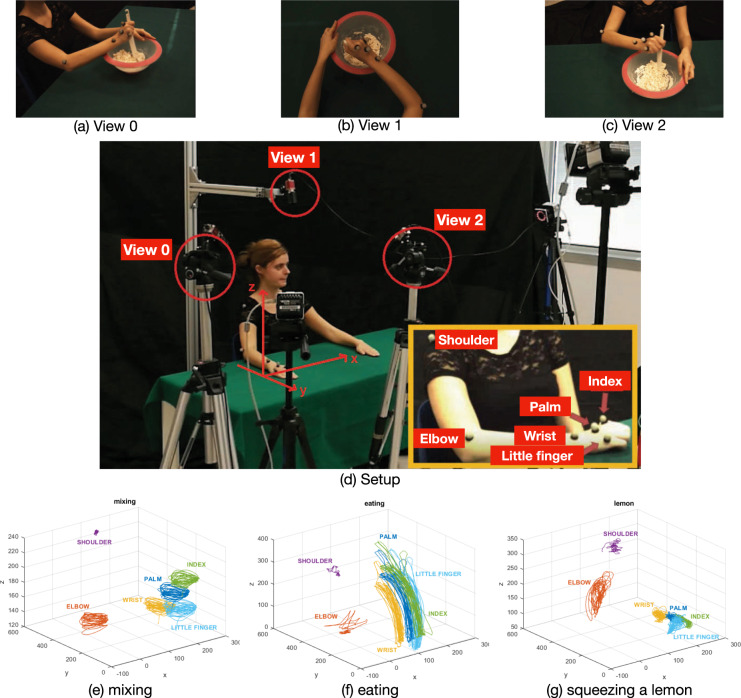
The acquisition setup (**d**) is composed by a table on which the subject performs a selection of cooking activities. The scene is observed with 3 cameras, placed in 3 different viewpoints (see **a–c**) and a motion capture system, collecting trajectories of joints positions over time (see in **e–g** examples of 3 different actions). The volunteer A. Vignolo gave the consent to include her photographs in the publication.

As shown in Fig. 1, we set up a simple working scene composed by a table covered by a uniform table cloth, a chair in front of it, and a selection of real cooking tools. We focus on upper body motion: during the acquisitions, the actor is sitting in front of the table, performing cooking activities that may, or not, involve the use of tools. The acquisition infrastructure is composed by three identical IP high resolution cameras, acquiring at a rate of 30 fps, and a Motion Capture (henceforth referred to as MoCap) system, composed by six VICON infrared cameras, acquiring at a rate of 100 Hz/s. This allowed us to acquire, synchronously, multi-view videos of the actor and the skeleton description of the moving arm.

The cameras are mounted on three tripods so that in all acquisitions we have a still uniform background and moving foreground objects. The cameras observe the scene from three different viewpoints: a frontal view (see Fig. 1c), a lateral view (see Fig. 1a), and a quasi egocentric view, obtained by a camera mounted slightly above the subject’s head (see Fig. 1b). In this way all the cameras are fixed, including the egocentric one. As for the MoCap, each infrared cameras is equipped with an infrared strobe, capturing the light emitted by six reflective markers placed on relevant joints of the right arm of the actor: shoulder, elbow, wrist, palm, index finger and little finger (see the details in Fig. 1d). Notice that the use of both arms for performing a certain task can be only appreciated in videos, since the reflective markers have been placed on the right arm.

The MoCap system has been calibrated so that all markers positions are expressed according to a common reference system, whose ideal origin is placed on the middle-left part of the table (see Fig. 1d). The positions are expressed in millimetres. Such positions, collected over time, form trajectories associated with the anatomical points corresponding to the marker location on the actor body. As shown in Fig. 1e,f, for actions *mixing*, *eating*, and *squeezing a lemon* respectively, joints dynamic provide different amount of information and importance depending on the specific action.

During the recordings, no specific constraint has been imposed to the volunteer, that has been instructed to act as naturally as possible, with the only attention to move within the boundaries of the working space. The actor performed 20 repetitions of the 20 actions reported in Table 2. Depending on the nature of the action, this corpus of data may result in a different number of action instances, as it will be discussed in the section discussing data annotation. To enable the use of the data in a Machine Learning framework, training and test sequences have been acquired in specific acquisition sessions.

In addition, we also acquired sequences of more structured kitchen activities, composed by sequences of the considered actions, to simulate different intermediate stages of the preparation of a meal. A main goal in this case was to consider more real situations, when different primitive gestures or actions are performed in sequence, with a smooth transition between them and with an inevitable influence on each other. The specific sequences – a summary of which is provided in Table 3 – have been designed so to offer different complexities: so if **Scene 2** is a slightly more complex variation of the grating a carrot action, the **Scene 4** has a more complex structure, being composed by a sequence of diverse primitive gestures and sub-goals, and with multiple objects involved in the manipulation.

**Table 3:** Sequences of actions for the 5 available scenes.

**Scene 1**	**Preparing an omelet:** beating eggs, reaching (the salter), transporting (the salter), salting, transporting (the salter), reaching (the jug), single pouring, transporting (the jug), reaching (the bowl), beating eggs
**Scene 2**	**Grating cheese:** reaching (the cheese), transporting (the cheese), grating (the cheese, corresponds to grating a carrot), transporting (the cheese)
**Scene 3**	**Melting ingredients** reaching (the bottle), transporting (the bottle), reaching (the lid), opening a bottle, transporting (the lid), reaching (the bottle), single pouring, reaching (the bowl), transporting (the bowl), mixing
**Scene 4**	**Making a sandwich:** cutting bread, transporting (the knife), transporting (the slice of bread), reaching (the knife), transporting (the knife), spreading, transporting (the knife), reaching (the slice of bread), eating
**Scene 5**	**Preparing a lemonade:** reaching (the lemon), transporting (the lemon), squeezing a lemon, transporting (the lemon), reaching (the squeezer), transporting (the squeezer), reaching (the cloth), transporting (the cloth), cleaning the table

The dataset finally includes about 1 h 35′ of acquisitions, of which 44′ are for the training sequences, 40′ are for the test sequences, and 11′ are for the scenes sequences.

We finally mention that MoCA is meant to be a growing project: we are planning new acquisitions covering a wider range of actions performed by different actors, possibly observed from new viewpoints. This will further expand the share of potential users, opening to new research questions and applications.

### Data cleaning

The sequences of markers positions acquired with the MoCap system may be affected by two main types of problems:

1. Reflections due to the surface of tools manipulated in certain actions may cause the presence of “dummy” markers, i.e., false detections
2. Temporary failures in markers detection may cause incomplete trajectories.

While for the first issue there is not a straightforward solution, to cope with the latter problem, the VICON software (https://www.vicon.com/software/) offers the opportunity to approximate the position of missing markers with a cubic spline interpolation.

No further filtering has been applied to the raw data.

3D coordinates have been exported in c3d files, the standard file format for MoCap systems acquisitions.

### Data synchronization

At the beginning of the recording, to facilitate the initial synchronization between the motion capture and the cameras streams, the actor performs an encoded action (snapping the fingers), that favours the manual identification of the initial times ${t}_{0}^{M}$ and ${t}_{0}^{V}$ of the motion capture stream and videos streams respectively.

Our two sources of data are not directly comparable: as mentioned above, video data are recorded with a sampling rate of 30 fps, while MoCap data are acquired at a sampling rate of 100 Hz/s, meaning that we can not find an exact discrete mapping between them; indeed, given a video frame timestamp *t*_*v*_, the corresponding *t*_*m*_ on the motion capture sampling is:

1 $${t}_{m}={t}_{0}^{M}+\left({t}_{v}-{t}_{0}^{V}\right)\ast 100/30$$

which would correspond to a discrete value (leading to an exact synchronization) every 0.5 seconds, corresponding to intervals of 15 video frames and 50 MoCap frames respectively.

Hence, given the synchronized starting instants of the two visual streams ${t}_{0}^{M}$ and ${t}_{0}^{V}$ (annotation provided, it can be found in file synchindex.csv), the rest of the synchronization can be obtained using the following formula:

2 $${t}_{0}^{V}+15\ast t={t}_{0}^{M}+50\ast t\;for\;t\in 0,T$$

where *T* depends on the length of the specific action.

### Data annotation

Data annotation concerned the identification of action instances in training and test sequences, and the detection and labelling of different actions in the scene sequences. The annotation has been mainly based on the analysis of the MoCap streams, while using the videos for a visual feedback. Two volunteers contributed to the annotation. More precisely, for each action sequence, we analysed the spatial trajectory covered by the marker placed on the palm of the hand, as it provides a stable characterization of the movements for all actions. From the trajectory, we identified meaningful cut points of the marker position along the most significant axis with respect to the MoCap reference system, marking the end of the portion associated with an action instance. Since the sequences are composed as repetitions of a same action, the principal axis is[are] the one[s] showing a repetitive pattern, facilitating the identification of cut points since they correspond to local extrema of the trajectory.

**Fig. 2:**
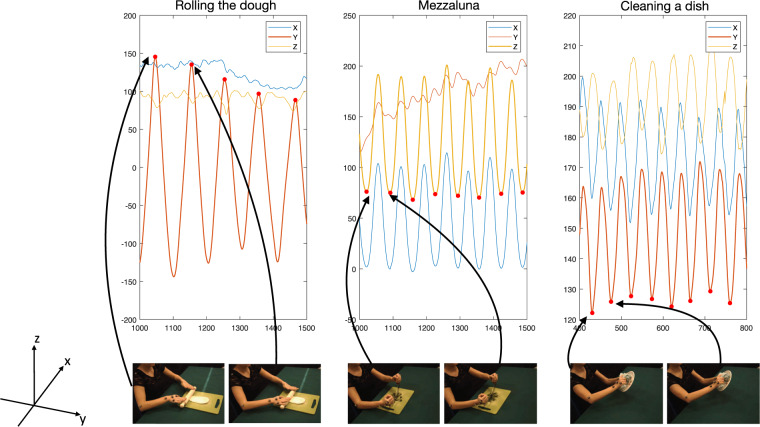
A sketch of the strategy followed for the annotation. The plots report the evolution of the 3 coordinates of different actions (*rolling the dough*, *mincing with the mezzaluna*, and *cleaning a dish*), and, marked with red circles, the time locations that have been manually annotated as action instance delimiters. Below, samples frames from View 0 clarify to which moment in the action the instants correspond to.

In Fig. 2 we report some examples to provide a visual intuition on the procedure: we show 3 actions observed from the viewpoint 0, together with the plots of their x, y, and z coordinates over time. We mark the annotated time instants with red dots. We notice the procedure above corresponds to mark the local maxima of the y-coordinate for *rolling the dough* (left), the local minima of the z-coordinate for *mincing with the mezzaluna* (middle), and the local minima of the y-coordinate for *cleaning a dish* (right).

Table 4 provides a visual impression of the action instances distribution for training and test sequences after annotation. The percentages are computed over the total number of action instances annotated on the training and test set respectively (516 for training and 610 for test). The absolute number of instances per action is reported in Table 2 (third column), along with a statistical analysis which will be discussed in the next section.

**Table 4:** Actions instances distribution in training and test sequences, after manual annotation (see Section *Data Annotation*).

	Action	Training	Test
1	Shred a carrot	12%	15%
2	Cut the bread	4%	3%
3	Clean a dish	4%	3%
4	Eat	3%	3%
5	Beat eggs	15%	16%
6	Squeeze a lemon	4%	3%
7	Mince with a crescent	5%	7%
8	Mix in a bowl	3%	4%
9	Open a bottle	4%	3%
10	Turn the pancake	4%	3%
11	Pestle	5%	4%
12	Pour water in containers	2%	2%
13	Pour water in a mug	4%	4%
14	Reach an object	4%	4%
15	Roll the dough	5%	4%
16	Wash the salad	4%	4%
17	Salt	3%	3%
18	Spread cheese on bread	4%	4%
19	Clean the table	4%	4%
20	Transport an object	7%	7%

As it can be noticed, for each action, a comparable amount of samples is available for training and test. Some actions are associated with a significantly higher number of samples. This holds true in particular for repetitive actions, as *mixing* or *beating eggs*, for which we decided to provide the annotation at the level of single gesture rather that at the level of action instance, as the specific number of gesture primitives composing the action instance is, for the nature of the action, variable and somewhat subjective. The hand-made decision of a specific splitting would have introduced a certain degree of bias in the annotation.

Concerning the annotation of the scenes sequences, we followed a similar procedure based on the analysis of the trajectory. In addition to the labelling of known actions, we also provide information on the presence of portions of the sequence which do not correspond to a known concept (they may be for instance related to a pause in the activity or the instance of an unknown action).

## Data Records

The dataset is publicly available on the GitHub (https://github.com/nicolettanoceti/cookingdataset) and figshare^19^ repositories. In this section we briefly introduce the data structures we devised to store and represent the MoCA dataset, and the functions for loading, visualizing and managing the dataset. The reference language is MATLAB, MathWorks, Inc.

### Data

The Cooking Dataset includes data regarding 20 different actions, as in Table 2. MoCap data streams and video recordings can be found respectively in folders |*data*/*mocap*| and |*data*/*video*|, both composed by a training and a test set, with a separate folder containing test scenes. In the figshare repository, the data are organized in a slightly different way, with no DATA folder. Moreover, for each action a folder (named accordingly to the action itself) contains two sub-folders with training and test videos. MATLAB structures containing the MoCap streams are composed by the following fields:

- **Shoulder, elbow, wrist, palm, index finger and little finger**: full raw MoCap streams, with no post-processing; see Fig. 1d.
- **index**: array of manual segmentation indices;
- **labels** (present only in the scenes structures): array of labels of the actions performed in the sequence. Labels include an additional pause label, associated with moments in the sequence in which the actor is idle.
  The point of view of the video recording is specified by the number at the end of each filename:
- “_0.avi” looks at the scene from the right, see Fig. 1a;
- “_1.avi” looks at the scene from the actor point of view, see Fig. 1b;
- “_2.avi” looks at the scene from the front, see Fig. 1c.

Loading and visualisation functions to access RGB and Kinematic streams can be found in the folder |*publicfunctions*|, along with a pdf file reporting data information.

## Technical Validation

In this section we first provide an experimental assessment of the data, then we explore possible scientific questions to be addressed with our dataset.

### Data assessment

For clarity, in this section, we consider one joint only. We select the *palm* as it is, on average, the most representative across actions of different granularity.

We start discussing simple evidences that can be derived by a preliminary data exploration on the Mocap sequences. A first characterization may be derived directly from the length of the action instances.

**Fig. 3:**
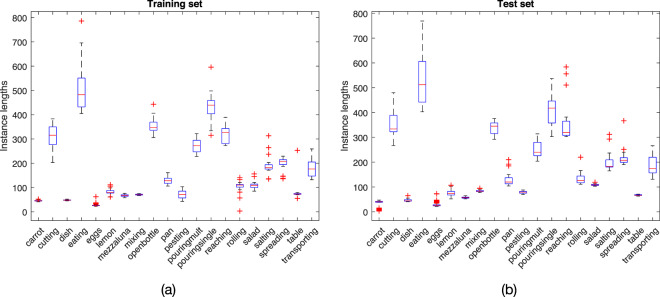
Lengths of action instances in training (left) and test (right) streams.

In Fig. 3 we notice how variance in times of execution is considerably higher for activities that require more than 2 seconds (that is, 200 frames) to be completed. These activities, in general, are more structured, include pauses and a higher displacement; examples are cutting the bread, eating, pouring water, reaching for an object, transporting an object.

**Fig. 4:**
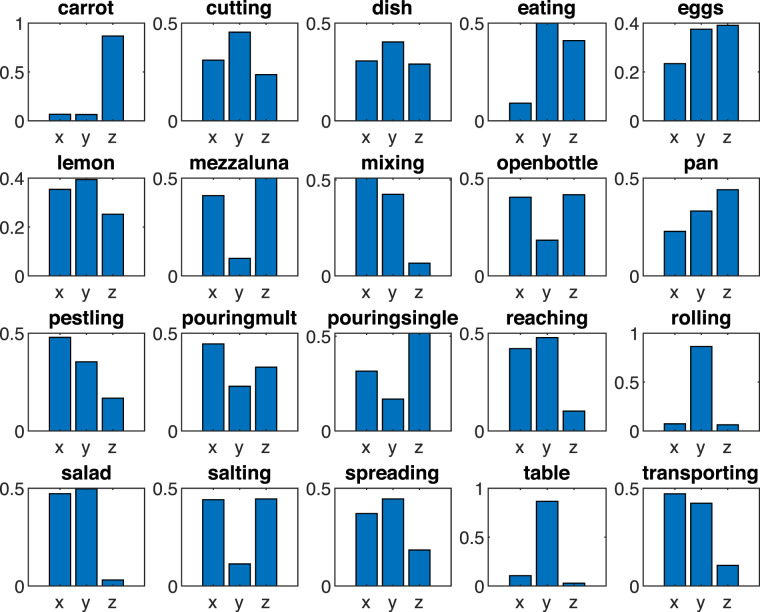
Normalized standard deviation of the palm 3D position for each action, referring to columns 4, 5 and 6 of Table 2. This visualisation emphasizes the presence of one or more peaks in the standard deviation of the 3 coordinates, suggesting a possible categorization of the actions – according to the number of dimensions in which the movement mainly evolves – and providing a guide for manual annotation.

Some of these considerations can be confirmed with a further data exploration we derived from an analysis of 3D positions. It provides a complementary actions characterisation to the one we initially provided in Table 2: if there, we highlighted ideal, objective properties guided by the action types and their goal, here we consider in Table 5 a quantitative assessment strongly related with the specific actions realisation and the data annotation we provided. More specifically, we compute standard deviations of positions and velocities (estimated as displacement between consecutive positions) along the three main axes for each action instance, in order to have an intuition on the main direction and spatial displacement of the movements. We may notice different categories of actions can be identified according to these spatial properties: for instance *carrot* and *rolling* mainly evolve along main direction, while in the case of *mixing* the important directions are two. In order to have an overall intuition on this aspect, we report in Fig. 4 a visual representation of these quantities, in terms of the standard deviations of the 3D positions for all the classes, normalized between 0 and 1 to facilitate the visual comparison.

**Table 5:** Some statistics and features on the annotated action instances.

Action		rep.	Std.Dev. 3D Pos.			Std.Dev. 3D Vel.			Vel. norm
#	name		X	Y	Z	X	Y	Z	
1	Shred a carrot	139	2.85	2.73	36.77	0.66	0.48	5.14	5.00
2	Cut the bread	35	16.68	24.38	12.69	1.34	2.43	0.76	2.54
3	Clean a dish	35	12.23	16.11	11.59	1.65	2.13	1.56	2.66
4	Eat	28	20.49	113.11	92.89	0.45	1.97	1.66	0.51
5	Beat eggs	157	3.79	6.07	6.33	0.98	1.60	1.51	2.41
6	Squeeze a lemon	40	9.31	10.36	6.63	0.89	1.06	0.65	1.49
7	Chop with a crescent	61	32.76	7.14	39.91	3.20	0.74	3.82	4.63
8	Mix in a bowl	37	33.68	28.12	4.37	3.00	2.56	0.43	3.90
9	Open a bottle	34	19.35	8.78	19.97	0.83	0.52	0.71	0.94
10	Turn the pancake	37	9.97	14.53	19.97	0.75	1.32	1.80	1.51
11	Pestle	43	21.65	15.99	7.58	2.04	1.66	0.89	2.87
12	Pour water in containers	22	18.04	9.25	13.22	0.49	0.26	0.43	0.42
13	Pour water in a mug	40	52.76	28.16	86.22	0.99	0.52	1.49	0.81
14	Reach an object	39	108.80	123.43	26.22	2.78	3.25	1.51	0.56
15	Roll the dough	47	6.66	78.90	5.78	0.55	4.58	0.64	5.27
16	Wash the salad	41	19.81	20.88	1.29	1.22	1.24	0.10	1.91
17	Salt	35	36.89	9.44	37.25	3.24	0.76	3.26	2.72
18	Spread cheese on bread	43	13.55	16.28	6.73	0.51	0.54	0.43	0.67
19	Clean the table	40	8.93	73.42	2.42	0.91	6.15	0.28	6.82
20	Transport an object	73	117.59	105.58	26.27	2.17	1.96	1.34	0.86

From left to right: *(Column 1):* actions identification number. *(Col. 2):* action name or description. *(Col. 3):* number of action instances manually annotated. *(Col. 4-5-6)*: standard deviation of the motion capture palm marker 3D positions, with respect to the three main directions (X, Y, Z). *(Col. 7-8-9):* standard deviation of the 3D velocities, computed from the motion capture palm marker position, with respect to the three main directions (X, Y, Z). *Col. 10*: 3D velocity norm.

**Fig. 5:**
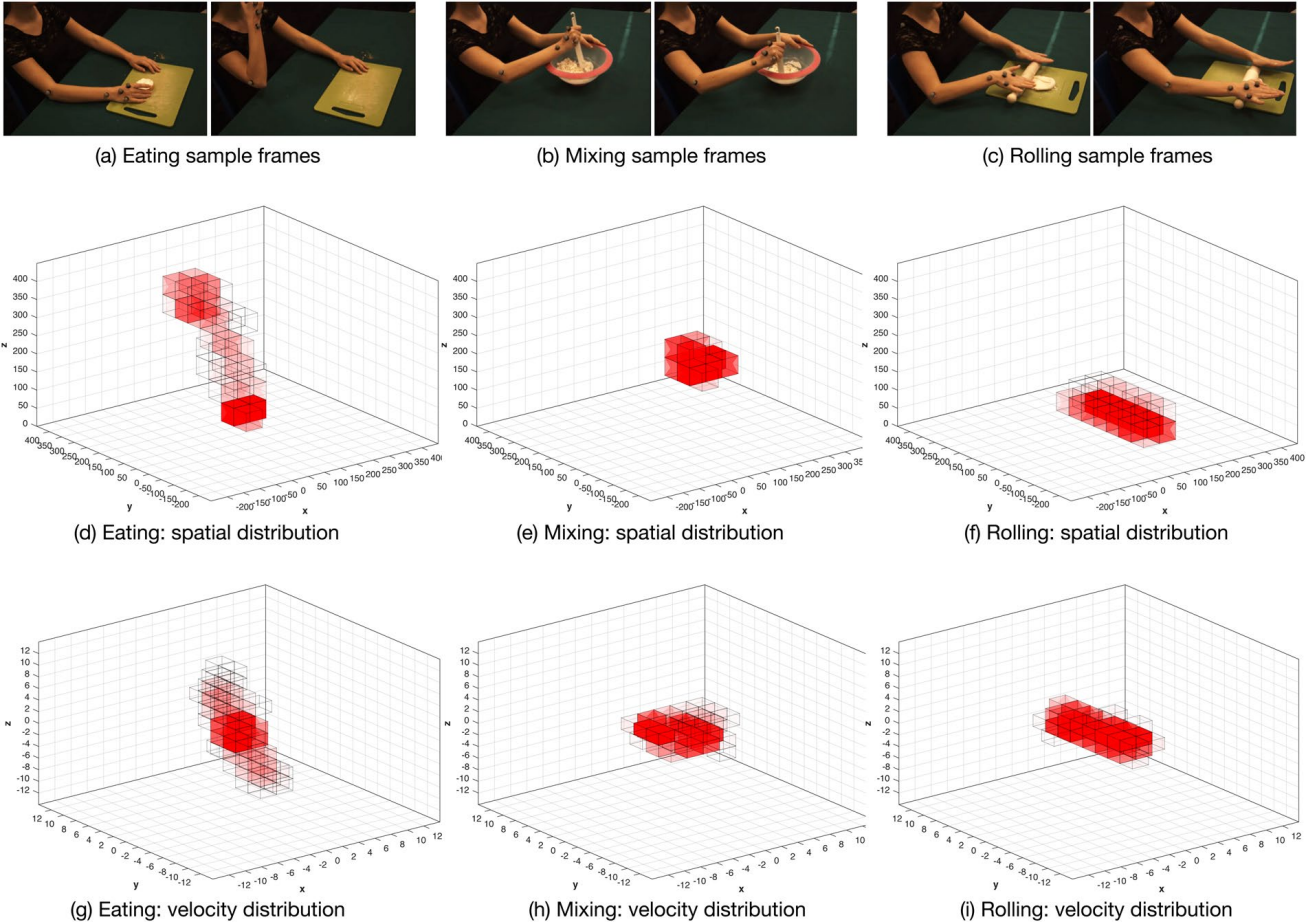
Example of 3D + t histograms for 3 different actions. Above: sample frames to show the evolution of actions. Middle: histograms of action positions. Below: histograms of instantaneous velocities. All histograms refer to the *palm* joint.

With an alternative visual representation, in Fig. 5 we also report sample frames from 3 actions and represent the evolution of 3D positions and 3D velocity by means of 3D + t histograms, computed by quantizing the appropriate feature space (positions and velocity) in equally spaced portions per input dimension, and then shown with a cube-based visualization. The frequency is inversely proportional to the transparency value in the visualization we propose. In the middle row, we show actions spatial distribution, in the bottom row their instantaneous velocity distributions. Meaningful peculiarities derived from simple motion properties of each action can be appropriately encoded with this visualization: the spatial distribution captures relevant trajectories (for instance the eating path), while velocity distribution highlight variations in the direction and magnitude of motion (notice how eating is a characterized by a uniformly low velocity, while mixing shows visible and smooth changes in direction, and rolling very relevant velocity peaks).

### A baseline analysis

In this section we provide a baseline analysis for the action classification tasks, some results already presented in^20^. We consider here the dataset portion of single actions and carry out action recognition with unimodal data. As for MoCap data, we adopt different combinations of the 3D + t histograms visualized in Fig. 5 (space only, velocity only, a concatenation of both) to model the space-time evolution of an action. To increase their effectiveness in characterizing the action, we concatenated the histograms related to 4 joints (elbow, wrist, palm, index) we empirically found to be the most important to the purpose. The obtained descriptions are finally used to feed a linear SVM classifier.

As for the video sources, here we proceed one view at a time (training and testing performed on the same view). To learn the representation we consider a variant of the Inception 3D^21^, a two-stream Inflated 3D ConvNets model, originally including two streams, RGB and Optical flow, jointly combined with a late fusion model. Conversely, we use only the flow stream of the network, also less prone to overfitting. The model is pre-trained on ImageNet dataset^22^ and on Kinetics-400^23^. Once trained, the network may be seen as a multi- resolution representation of image sequences. The features learnt from the optical flow are flattened, and after a random dropout they are fed into a classifier. We compared two different strategies: the use of a Single Layer Perceptron (SLP) followed by a batch normalization layer, to promote regularization of the solution, and the adoption of the full original architecture, as in^21^. Results are reported in Table 6, that also includes a result from a state-of-art method on action recognition from skeleton data^24^. The regularity of the movements that the volunteer attains when performing repeatedly the very same task favours the overall uniformity of the replicas, thus facilitating the classification despite the actions complexity.

**Table 6:** Action recognition benchmark, see text. (*) The final 2 layers of the *I*_3_*D* model were finetuned on the training data.

	Method	Accuracy
MoCap	Space 3D + *t* histograms + linSVM^20^	0.92 ± 0.19
	Vel 3D + t histograms + linSVM^20^	0.82 ± 0.27
	Full 3D + t histograms + linSVM^20^	0.95 ± 0.11
	Haskel^24^	0.98 ± 0.01
Videos	*I*_3_*D* features + SLP	0.94 ± 0.22
	Full *I*_3_*D* model(*)	0.92 ± 0.23

### Examples of use of the MoCA

We now discuss some examples of scientific questions that can be profitably explored using the MoCA dataset as a test-bed. Noticeably, the tasks refer to different domains, ranging from Cognitive Science to Computer Science, with applications to Human-Human Interaction and Human-Machine Interaction.

#### Selecting action timing for collaborative Human-Machine Interaction

To work efficiently together, human and artificial agents require a mutual understanding of what the partner is doing and when it is the right moment to act. To this purpose action segmentation allows the artificial agent to understand when an action is ending. In some cases one may also identify finer relevant time instants, within an action, which may be informative of the partner’s movement timing. The instants can be interpreted as temporal locations where the time signal describing a motion can be ideally segmented, providing a set of primitives that can be used to build a temporal signature of the action and finally support the understanding of the dynamics and coordination in time. Such relevant instants can be detected by exploiting motion information embedded in the so-called *dynamic instants*, *i.e*., time instants in which the dynamic of an action is subject to a change, that may be due to variations in velocity, acceleration, or direction of motion. In^25^, dynamic instants are identified as minima of the velocity profile, directly derived from the optical flow^26^, and then classified as instants where an action is starting, ending, or changing^27^.

**Fig. 6:**
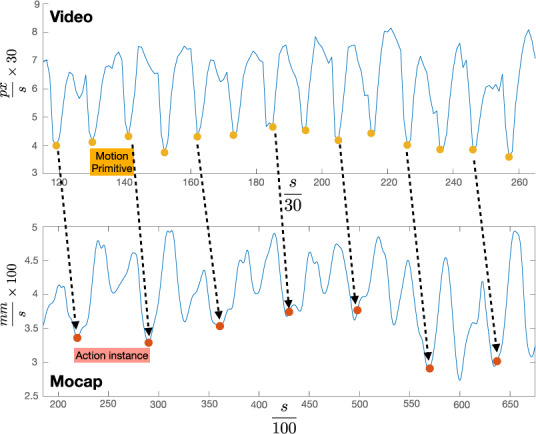
A visual comparison between time locations corresponding to dynamic instants – i.e. local minima of a velocity profile obtained from optical flow maps as in^25^ – and to the annotation we provide for the MoCA for a sequence of *mixing* actions. While the latter identifies action instances, the first delimit motion primitives.

In Fig. 6 we show how the dynamic instants in^25,27^ relate with the annotation we provide for the MoCA (for the plots we considered a *mixing* action). The two plots – referring to a video (top) and a Mocap (bottom) sequence – have been adjusted to consider the same temporal extent, using Eq. 1. While the annotation identifies action instances (a full mixing round), the dynamic instants delimit motion primitives (the two halves of the round). Detection and annotation can thus be jointly used to reason on actions and their decomposition in sub-structures. Furthermore a similar approach has proven to support the possibility for a robot to coordinate in time with human partners performing a repetitive action, with no a priori knowledge of the spatial properties of their movements^27^.

#### Context-less human judgments of motion similarities

An open research question refers to understanding how humans perceive actions and their similarities. Investigating what are the features that we consider when we have to judge whether two movements are two instances of the same action, even in absence of contextual information, may provide useful insights on how to enable the same capabilities on artificial agents and establish a shared perception with them.

**Fig. 7:**
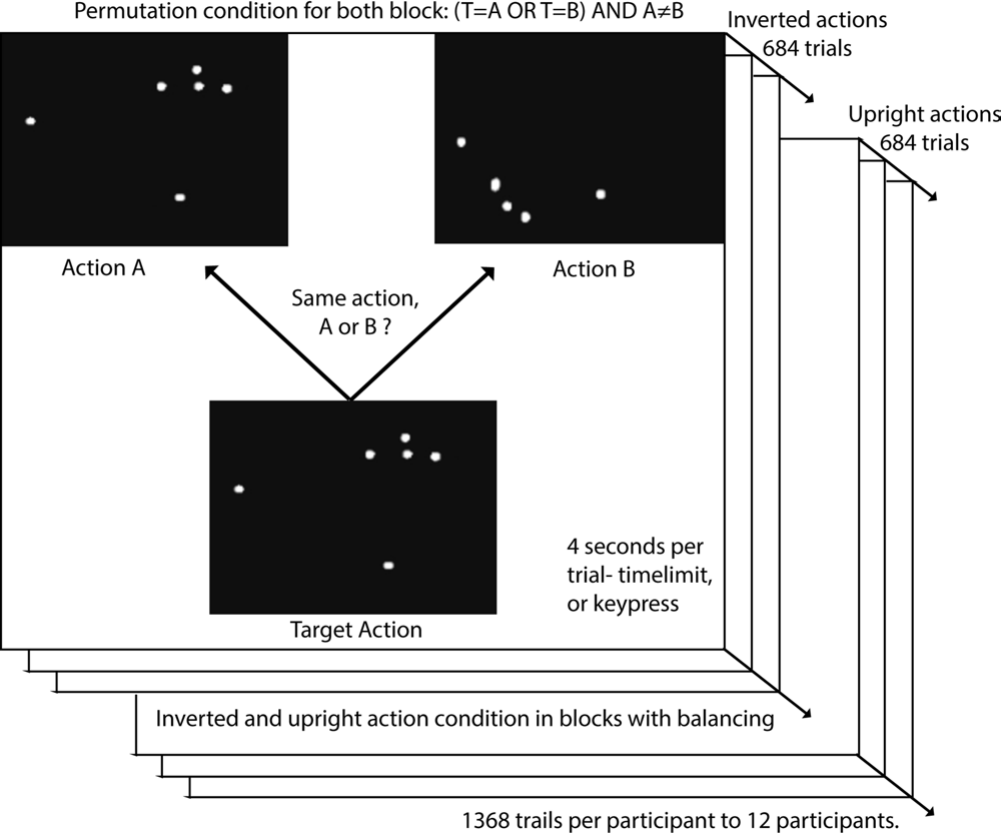
A visual representation of the experiment designed to evaluate the ability of humans to judge action similarities in absence of contextual information.

The MoCA dataset can be used to this purpose. In Fig. 7 we provide a visual representation of an experiment in which the actions are provided to users with no contextual information (using *visualizeSkeleton* to show the skeleton over time), asking to judge the similarity among different action instances. The experiments, that have been described in^28^, have been organized as follows. Triplets of instances of actions (that we will call stimulus A, stimulus B and Target Action) have been concurrently shown to users, that had to decide whether the Target was of type A or B.

With the aim of understanding the relationships, if any, between a biological agent and an artificial one, an analogous experiment has been replicated using the computational models described in^20^. The outcome of the analysis suggested that, overall, human visual system seems to prioritize the spatial properties of the visual stimuli (including their relative positions) more often than the speed information^29^.

This observation opens to further interesting questions, related to the relative importance of the kinematic features depending on the conditions of observations of the motion, for instance the viewpoint or the specific type and granularity of the movements. In this sense, the MoCA represents an ideal test-bed.

#### Cross-view action recognition

As a last example, we consider view-invariant action recognition. This task plays a crucial role in humans, supporting the capability to solve the correspondence problem, i.e., identifying a mapping between the others’ actions and their own, which is necessary for crucial activities as social learning, imitation or mimicry^30^.

In this section we report the results of an analysis based on a domain adaptation procedure that allows us to counter-balance the limited size of MoCa with respect to the typical size required by modern machine learning architectures. Specifically, we assess the potential of pre-trained intermediate deep features in mimicking the role of view-dependent neurons and view invariant higher level descriptions. The features are the same we adopted in the baseline analysis. The resulting validation accuracies are shown in Table 7. We first consider a *one-view out* protocol, when the classifiers are trained with 2 viewpoints and tested on the third; if compared to baseline results (see Table 6), there is a notable and expected drop in the capability of the classifiers to correctly classify the actions, but considering they are not explicitly trained to identify actions view-invariantly, this drop is not remarkable. Notice in particular how the egocentric view is the hardest to classify if it does not participate in the training phase. Second, we adopt a *one-one* protocol, training classifiers on a single viewpoint and evaluating on another viewpoint, to analyse view-view relationship. When both views are allocentric ({0|2},{2|0}), the resulting values are almost as high as in one-view out experiments. But in all cases where V1 is involved in the one-one protocol ({0|1},{1|0},{1|2},{2|1}), there is a noticeable drop in the performance. The results highlight the specific challenge in dealing with view invariance when ego-vision is one of the views considered. This experiment raises further multidisciplinary questions that could be explored with the help of the proposed dataset. Indeed, from the numerical point of view, the consideration on the complexity of ego-vision appears to be understandable for the smaller amount of dynamic information included in the ego view of the actions. Instead, it is in contrast with recent findings in neuroscience. First-person view seems to have a prominent role relative to other perspectives in terms of responsiveness in the sensorimotor areas of the brain during action observation^31^ and has been shown to facilitate certain forms of action understanding (e.g., estimating the size of an object to be grasped)^32^.

**Table 7:** Performance evaluation (in %) on the MoCA dataset considering various training and test subsets. Views - 0: Lateral, 1: Egocentric, 2: Frontal.

Source|Target	0,1|2	0,2|1	1,2|0	0|1	0|2	1|0	1|2	2|0	2|1
SLP	67.46	46.03	68.10	47.38	68.33	47.38	32.86	66.27	34.84
Inception	62.30	61.67	62.70	50.63	64.84	33.10	36.35	61.67	54.92

#### Marker-less pose estimation

**Fig. 8:**
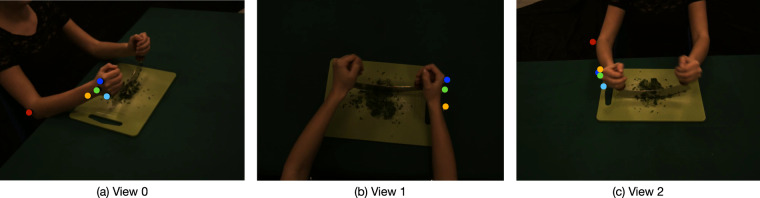
Sample frames to show the potential of a marker-less analysis for feature detection. The localized points (highlighted with different colors in the images) are nicely overlapped with the markers placed on the arm, that the method has been trained to detect.

We close the section with a view on our preliminary results in the domain of motor control. We are exploring the use of state-of-art marker-less methods for human pose estimation^33^ and motion analysis, as opposed to a marker-based approach, the gold-standard in in motor control applications. In Fig. 8 we show examples of features automatically detected on the videos, with a detection method based on the DeepLabCut architecture^34^, from where the accuracy in the detection – regardless the specific viewpoint – can be appreciated. The MoCA, providing both videos and motion capture data, is an ideal test-bed for a quantitative comparison between the different strategies of detection.

## Data Availability

We made available the following functions that allow the user to load and process the data. Further information on how to use the code can be found in the correspondent MATLAB files. • *loadDataset, loadAction*: allow the user to load and save the MoCA dataset in an easy-to-use data structure. *loadAction* gives the user the possibility of loading only part of the data streams, specifying for example an action label, a marker or an instance; • *segmentAction*: segments the single instances of action from the full MoCap streams. It makes use of the **i**ndex array mentioned above. Three types of visualisation functionalities are available: • *visualiseAction*: it produces a 3D plot of each marker’s trajectory • *visualiseSkeleton*: shows the arm skeleton over time while performing a complete action instance, from MoCap data; • initSynch and synchronizedView: jointly shows the action using RGB and Kinematic data. By means of the csv file synch_index.csv, the function *initSynch* prepares the data structures used in **synchronizedView** for the actual visualisation. All the functions we provided can be used also on the test scenes.
